# Cement Kiln By-Pass Dust: An Effective Alkaline Activator for Pozzolanic Materials

**DOI:** 10.3390/ma11091770

**Published:** 2018-09-19

**Authors:** Lukáš Kalina, Vlastimil Bílek, Tomáš Kiripolský, Radoslav Novotný, Jiří Másilko

**Affiliations:** Materials Research Centre, Faculty of Chemistry, Brno University of Technology, Brno 61200, Czech Republic; bilek@fch.vut.cz (V.B.J.); xckiripolsky@fch.vut.cz (T.K.); xcnovotny2@fch.vut.cz (R.N.); masilko@fch.vut.cz (J.M.)

**Keywords:** cement kiln by-pass dust, alkali activation, blast furnace slag, admixture

## Abstract

Cement kiln by-pass dust (CKD) is a fine-grained by-product of Portland clinker manufacturing. Its chemical composition is not suitable for returning back into feedstock and, therefore, it has to be discharged. Such an increasing waste production contributes to the high environmental impact of the cement industry. A possible solution for the ecological processing of CKD is its incorporation into alkali-activated blast furnace slag binders. Thanks to high alkaline content, CKD serves as an effective accelerator for latent hydraulic substances which positively affect their mechanical properties. It was found out that CKD in combination with sodium carbonate creates sodium hydroxide in situ which together with sodium water glass content increases the dissolution of blast furnace slag particles and subsequently binder phase formation resulting in better flexural and compressive strength development compared to the sample without it. At the same time, the addition of CKD compensates the autogenous shrinkage of alkali-activated materials reducing the risk of material cracking. On the other hand, this type of inorganic admixture accelerates the hydration process causing rapid loss of workability.

## 1. Introduction

Worldwide, the material resource consumption increases and consequently large amounts of waste are released into the environment [1]. This phenomenon is also associated with Portland cement production. It is well known that cement production is responsible for approximately 7% of the world’s CO_2_ emissions [2]. Moreover, the manufacturing of Portland clinker is associated with solid waste production. The cement kiln by-pass dust (CKD), is the typical by-product of the cement clinker burning process [3]. This material contains high concentrations of heavy metals and therefore should be disposed as a hazardous waste [4]. Nevertheless, the economic pressure caused by CKD disposal tends to its re-addition to the final cement product.

A possible way to ecologically process the CKD waste is utilization in alkali-activated materials formed via the reaction of a solid aluminosilicate with highly concentrated alkaline aqueous solution [5]. The alkali activation mechanism includes the destruction of the raw material by breaking the Si–O–Si and Si–O–Al bonds into lower stability structural units, their interaction through the coagulation-condensation process and the precipitation of final reaction products [6]. Thanks to the alkaline contents of CKD, especially due to the free lime, the significance of its usage as an alkaline activator arises. Previous studies [7,8] show that the alkali activation process supported by reactive lime from CKD leads to the creation of calcium silicate binder phases. However, the pH value of alkaline solution with CKD is not high enough for sufficient decomposition of raw materials which in most cases need the addition of a typical alkaline activator such as sodium hydroxide [9,10]. In terms of working with such a caustic chemical compound, one must observe the safety regulations, which makes it more difficult in practical usage. Therefore, the study is focused on the alkali activation of blast furnace slag with CKD in connection with sodium carbonate resulting in sodium hydroxide according to Equations (1) and (2).

CaO + H_2_O → Ca(OH)_2_, (1)

Ca(OH)_2_ + Na_2_CO_3_ → 2NaOH + CaCO_3_, (2)

The caustic soda is formed gradually during the hydration process and increases the pH value in the pore solution which promotes the dissolution of aluminosilicate materials and thus positively affects the mechanical properties of the final products.

Thanks to the optimal chemical composition, the CKD seems to be an effective alkaline activator and plays a significant role in autogenous shrinkage mechanism in alkali-activated BFS materials. Therefore, CKD is a promising by-product material, and this paper is focused on its study and its possible utilization in these systems.

## 2. Materials and Methods

### 2.1. Materials and Sample Preparation

The main aluminosilicate material used to produce the alkali-activated mortars was blast furnace slag (BFS) with the Blaine fineness of 400 m^2^ kg^−1^ (ArcellorMittal Ostrava, a.s., Ostrava, Czech Republic). The cement kiln dust (CKD) was collected from the by-pass system of the Horné Srnie cement plant (Cemmac, a.s., Horné Srnie, Slovakia). The chemical composition of raw materials was determined by XRF as shown in Table 1. The XRD analysis of BFS indicated the presence of approximately 90% of amorphous phase determined with the method of internal standard (fluorite). The main minerals identified in BFS were melilite, calcite and merwinite. The CKD was composed of sylvite (KCl), lime (CaO), arcanite (K_2_SO_4_), larnite (2CaO∙SiO_2_), quartz (SiO_2_), portlandite (Ca(OH)_2_) and hatrutite (3CaO∙SiO_2_). The free lime content determined according to EN 451-1 was 27.2 wt %. The particle size distribution D50 of BFS and CKD determined by laser granulometry in dry state were ~10 µm and ~4 µm, respectively. Anhydrous sodium carbonate (Penta, s.r.o., Prague, Czech Republic) in the form of powder and sodium water glass (Vodní sklo, a.s., Brno, Czech Republic) with the silica modulus of 2.2 were used as the alkaline activators. The pH value of used sodium water glass was 12.48. The Na_2_O/BFS ratio was set to 8 wt %.

Alkali-activated BFS mortars with different amount of CKD at the expense of BFS (0; 5; 10; 15; 20; 25 wt %) were prepared. The sand-to-BFS ratio was 3:1 using three different fractions of standard siliceous sand meeting requirements of EN 196-1 and the water-to-BFS ratio was set at 0.46 with respect to the water contained in the sodium water glass. The composition of mortars is shown in Table 2. Mixing and curing processes in molds were carried out at laboratory temperature (25 °C) with RH~99%. After the demolding process (1 day), the specimens were stored in water at 25 °C in the curing chamber. Prepared samples were subjected to compressive, flexural strength determination and length change measurements.

### 2.2. Physical-Mechanical Measurements

The samples with the dimensions of 4 × 4 × 16 cm were tested to determine their compressive and flexural strength according to EN 196-1. The strengths were tested at the age of 1, 7 and 28 days. The measurements were carried out by the compressive and bending strength tester Betonsystem Desttest 3310. The length changes for obtaining the shrinkage-expansion evolution were measured in short time intervals using the ASTM C490 apparatus for 28 days. The consistence of fresh alkali-activated mortars was measured based on the mini-cone test procedure in accordance with EN 1015-3. The effect of gradual CKD additions on the pH in water solution with dissolved sodium carbonate was measured using pH-meter S213 SevenCompact Duo (Mettler-Toledo GmbH, Greifensee, Switzerland) until the pH value was constant (approximately 10 min).

### 2.3. Isothermal Calorimetry

The evolution of hydration heat was monitored using the TAM Air isothermal microcalorimeter (TA Instruments, New Castle, DE, USA). The measurements of heat evolution were performed at a constant temperature of 25 °C. When the thermal equilibrium was achieved, the BFS and alkaline activator were mixed together by injecting the solution into the 15 mL vial and stirring it for 3 min. The water/BFS, as well as the Na_2_O/BFS mass ratio, were the same as in the preparation process of mortars. The heat evolution was recorded as the heat flow immediately.

### 2.4. X-ray Diffraction Analysis (XRD)

The X-ray powder diffraction of alkali-activated matrices after 7 days of curing was measured using the PANanalytical Empyrean diffractometer with CuKα radiation equipped with a 3D detection system PIXcel3D. The specimens were step scanned from 5° to 40° 2θ using vertical high-resolution goniometer with the step size 0.013° 2θ. The samples with 0, 10 and 20 wt % of CKD were used for the determination of mineralogical composition after the hydration process, moreover for the verification of reaction mechanism outlined in Equations (1) and (2).

### 2.5. Thermogravimetry (TG)

The behavior of the alkali-activated matrices during the thermal treatment was investigated using 70 mg of the milled sample after 7 days of curing. The thermogravimetric analysis was performed using Q600 analyser (TA Instruments, New Castle, DE, USA) up to 1000 °C with the ramp 5 °C per minute and under the dried air conditions. The samples with 0, 10 and 20 wt % of CKD addition were used for the quantification of the created calcium carbonate, formed according to Equation (2).

## 3. Results and Discussion

**Figure 1:**
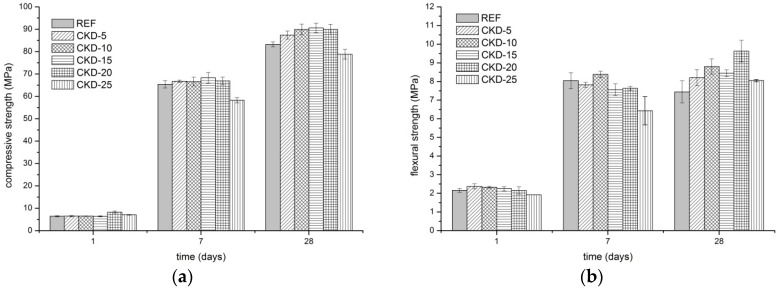
Compressive (**a**) and flexural (**b**) strengths development of alkali-activated mortars with different amounts of CKD.

**Figure 2:**
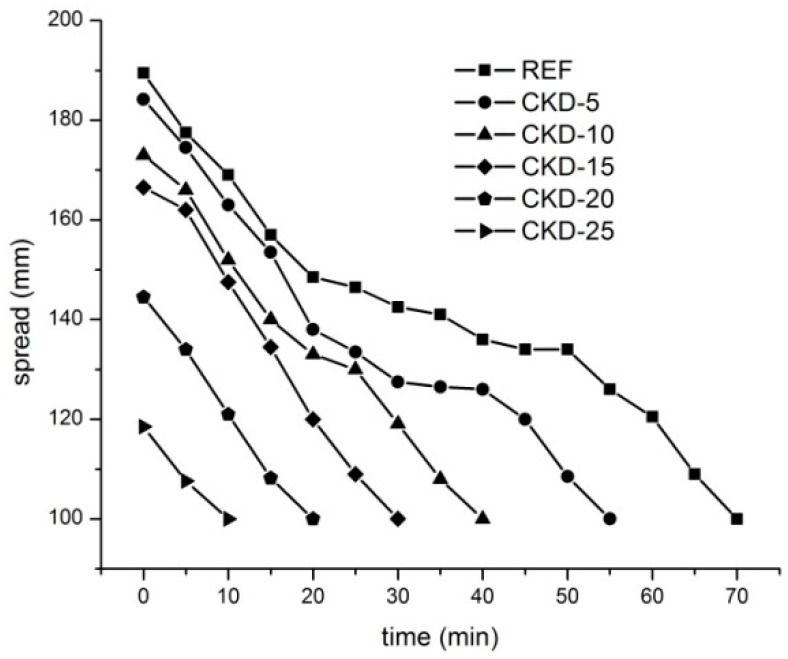
Workability over time of fresh alkali-activated mortars with different amount of CKD.

The compressive (Figure 1a) and flexural (Figure 1b) strengths development demonstrates a positive effect of CKD on mechanical properties. As observed in Figure 1a, the addition of CKD only slightly increases early compressive strengths after 1 and 7 days. However, a perspicuous improvement was achieved after 28 days, when all samples with the addition of CKD up to 20 wt % indicated higher strengths compared to the reference sample. Conversely, the substitution of 25 wt % BFS by CKD decreases the compressive strength which is related mainly to very quick loss of workability; this mixture is workable for only several minutes, as is shown in Figure 2. This strongly influences the homogeneity of samples.

Considering the measurement incertitude expressed by the error bars in all measurements, the flexural strength development after 7 days suggests a convincing increase in strength compared to 1-day-old samples. However, a significant difference is noticeable after 28 days of curing. Whereas the samples with CKD content show an increase in flexural strengths, the reference sample has the opposite evolvement. This phenomenon likely relates to the chemical shrinkage of pure alkali-activated BFS which is described in several studies [11,12]. As a result, the internal stress is present in the material and, therefore, the microcracks in the structure can take place, which negatively influence the flexural strength especially.

**Figure 3:**
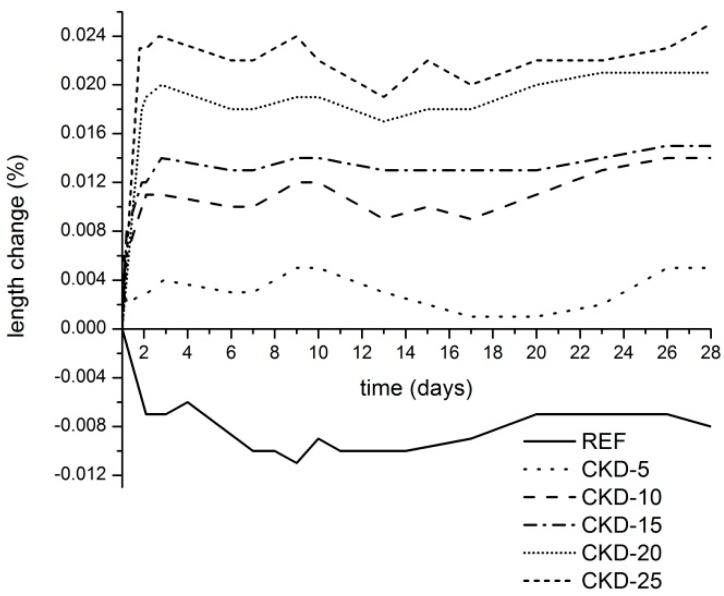
Length changes of alkali-activated mortars with different amounts of CKD during saturated (under water) curing.

The changes of samples length are shown in Figure 3. It is clearly visible that the reference sample exhibits the shrinkage which continues even after 1 day of mixing. This behavior in volume changes of alkali-activated BFS is in contrast with the binders based on ordinary Portland cements (OPC), since these binders cured continuously in water exhibit an increase in volume. This expansion, named as swelling, is connected with the absorption of water by the cement gel where the molecules of water act against cohesive forces and tend to force the gel particles away from each other [13]. On the other hand, the alkali-activated BFS forms CSH gel with the high atomic packing density which prevents water from penetrating into the inner structure during saturated (under water) curing [11] and causes self-desiccation. This process, which takes place in the interior of mortar mass, is known as autogenous volume change [13] and leads to the shrinkage of the whole system. A different situation occurs in the case of the samples with the CKD additions, the expansion of which depends on the amount of free lime contained therein. Free CaO is very well known as a cheap expansive agent, provided that it is burned at temperatures above 1000 °C resulting in “dead burnt lime” [14]. Such lime is also present in CKD, the collection of which from the Portland cement manufacturing process is carried out at the temperature of about 1100 °C. From the evolution of expansion it is obvious that the complete expansion is achieved in less than 3 days in all samples. This certainly has its advantages in the lower affecting of the curing process as well as in the lower risk of presence of residual un-reacted CaO which could cause later expansion [15].

**Figure 4:**
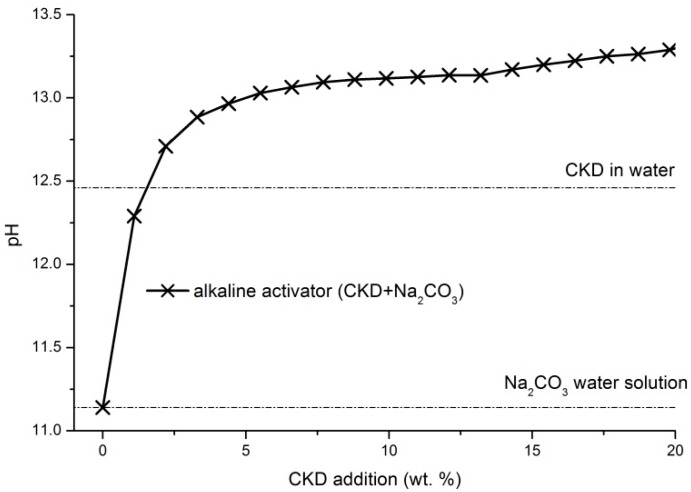
Effect of different amounts of CKD additions on the pH of water solution with dissolved sodium carbonate.

The increase in mechanical properties of samples with CKD addition is also closely connected with higher dissolution of BFS and subsequent higher formation of binder phases. The dissolution of slag particles strongly depends on the pH of ambient solution [16]. It is obvious that CKD as well as sodium carbonate mixed separately with water indicate lower values of pH in comparison with their joint solution (Figure 4). Thanks to the reaction of lime contained in CKD and sodium carbonate in the water environment, described in Equations (1) and (2), the rise in pH up to the value over 13 occurs and the dissolution of slag particles rapidly increases.

**Figure 5:**
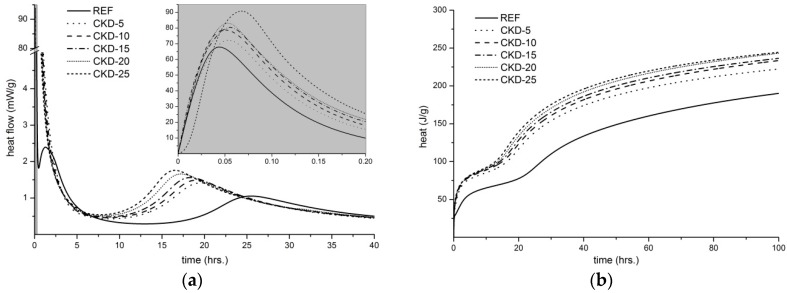
Evolution of heat flow (**a**) and total heat (**b**) of alkali-activated matrices with different amounts of CKD.

The process of dissolution of BFS and the creation of CSH gel was characterized through the isothermal calorimetry as shown in Figure 5. The first peak (Figure 5a), measured during the first minutes of hydration, is mainly associated with wetting and dissolution of BFS [17]. From the zoomed area it is clear that the samples with the higher content of CKD indicate higher heat flow which would confirm better dissolution of BFS particles. At the same time, the hydration of lime proceeds according Equation (1); therefore, the contribution of heat released by this reaction is also a part of the first peak. After that, the pre-induction band is observed belonging to the formation of primary CSH gel [18] and gaylussite (Na_2_Ca(CO_3_)_2_∙5H_2_O) which is one of the initial products in Na_2_CO_3_-activated slag binders [19]. The secondary formation of CSH gel is connected with the peaks from 10 to 40 hours. It is well observed that higher heat flow in this region was measured for the samples with a higher content of CKD. These results suggest that CKDs contribute to a larger formation of binder phase as proven by the measurement of the total amount of heat evolution (Figure 5b), which is particularly associated with the quantity of created CSH gel. The total heat was monitored within 4 days from the beginning of alkaline activation.

**Figure 6:**
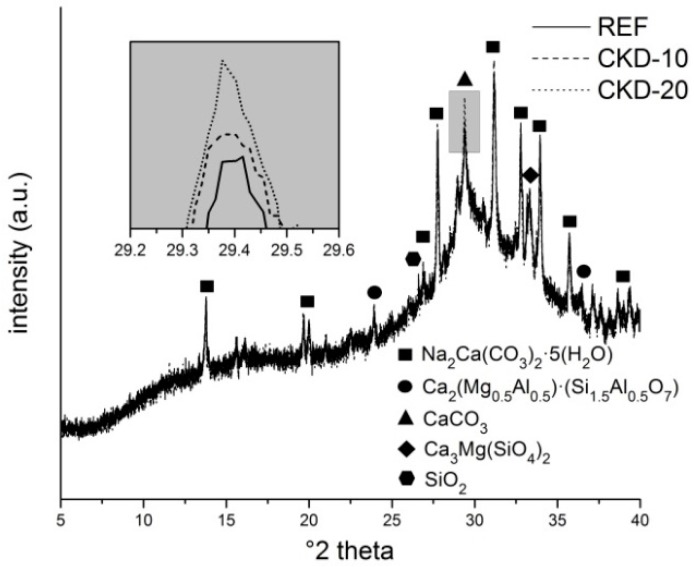
XRD of alkali-activated matrices with 0, 10 and 20 wt % of CKD addition.

Subsequently, the samples were subjected to XRD analyses (Figure 6). The overlapped diffractograms show the presence of minerals from the raw materials plus gaylussite. The comparison of mineralogical composition among the samples with different amounts of CKD indicates no significant changes except in the formation of calcium carbonate. The samples with higher content of CKD create a considerably higher quantity of calcite, resulting primarily from Equation (2).

**Figure 7:**
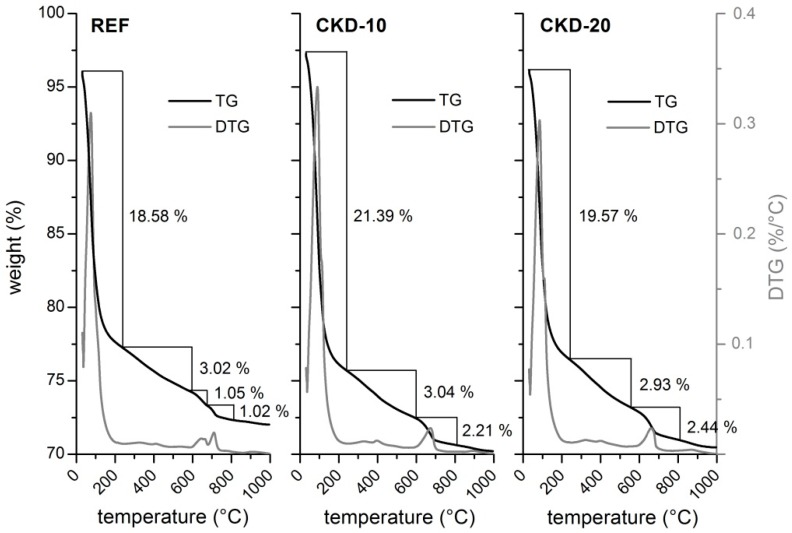
TG, DTG analyses of alkali-activated matrices with 0, 10 and 20 wt % of CKD addition.

The detailed quantitative information about the amount of formed calcium carbonate in prepared alkali-activated matrices was given by using of the thermogravimetric analysis. Figure 7 shows that the mass loss is connected with several ongoing processes during the heating. The first one between 31 and 240 °C can be associated with the loss of physically-bonded water and also the dehydration of chemically-bonded water from the newly formed CSH gel structure. The dehydration of interlayer water bonded in the CSH phase continues up to 600 °C [20]. The next process which appears around 600 to 800 °C can be attributed to the decomposition of carbonates. The first derivative of the TG analysis of the reference sample showed that the two step decomposition of carbonates takes place. The first step is supposed to be associated to the decomposition of gaylussite structure [21] and the second one to the calcite one [22]. The presence of both of them was confirmed by previous XRD analyses. However, it should be emphasized that the decomposition of the carbonate species should be intertwined together. The analyses of the samples with the CKD content shows the slightly different behaviour in this temperature range. It is obvious that the content of the gaylussite decreases with the expense of calcium carbonate, which was proven by the XRD analyses. From the conducted results we can conclude that the content of calcium carbonate increases with the reaction time. This phenomenon should be related with the carbonation process from some minor CO_2_ uptake onto the surface between the preparation of samples and TG analysis but it should also be a part of the reaction mechanism described in Equation (2).

## 4. Conclusions

The results of this study demonstrate that the secondary raw materials such as CKD can be successfully utilized for the alkaline activation of BFS. The following conclusions summarize the experimental part of the work:

1. The combination of CKD and sodium carbonate together with sodium water glass used as alkaline activators leads to the secondary formation of sodium hydroxide causing an increase in the pH of water solution in mixtures which promote the dissolution process of activated aluminosilicate.
2. Higher degree of BFS dissolution influences the hydration process in the sense of higher binder phase creation which positively affects the mechanical properties up to a certain limit.
3. The CKD content in alkali-activated BFS causes a small expansion of the whole system and thereafter the shrinkage cracking connected with the decrease of compressive as well as flexural strengths is reduced.
4. The addition of CKD into the alkali-activated systems decreases the workability due to early hydration of lime resulting in the system inhomogeneity which can strongly influence the mechanical properties.
5. The production of alkali-activated BFS binders with CKD addition depends on its optimum dosage into the system, moreover, the CKD chemical and phase composition must always be taken into account.

## Figures and Tables

**Table 1:** Chemical composition of BFS and CKD as determined by XRF.

Raw Material	Chemical Composition/wt %										
	SiO_2_	Al_2_O_3_	CaO	Na_2_O	K_2_O	MgO	SO_3_	Fe_2_O_3_	TiO_2_	MnO	Cl^−^
BFS	34.7	9.1	41.1	0.4	0.9	10.5	1.4	0.3	1.0	0.6	–
CKD	11.9	4.2	45.7	0.4	16.9	0.9	7.2	2.4	0.3	–	10.1

**Table 2:** Composition of alkali-activated mortar samples (wt %).

Mixture Designation	REF	CKD-5	CKD-10	CKD-15	CKD-20	CKD-25
BFS	21.6	20.5	19.4	18.3	17.2	16.1
CKD	–	1.1	2.2	3.3	4.4	5.5
Na_2_CO_3_	2.1	2.1	2.1	2.1	2.1	2.1
Na-water glass	3.5	3.5	3.5	3.5	3.5	3.5
water	8.0	8.0	8.0	8.0	8.0	8.0
standard sand	64.8	64.8	64.8	64.8	64.8	64.8
